# De novo copy number variations in cloned dogs from the same nuclear donor

**DOI:** 10.1186/1471-2164-14-863

**Published:** 2013-12-09

**Authors:** Seung-Hyun Jung, Seon-Hee Yim, Hyun Ju Oh, Jung Eun Park, Min Jung Kim, Geon A Kim, Tae-Min Kim, Jin-Soo Kim, Byeong Chun Lee, Yeun-Jun Chung

**Affiliations:** 1 Integrated Research Center for Genome Polymorphism, Department of Microbiology, The Catholic University of Korea, College of Medicine, 505 Banpo-dong, Seocho-gu, Seoul 137-701, Korea; 2 Research Institute for Veterinary Science and Department of Theriogenology and Biotechnology, College of Veterinary Medicine, Seoul National University, 1 Gwanak-ro, Gwanak-gu, Seoul 151-742, Korea; 3 National Creative Initiatives Research Center for Genome Engineering and Department of Chemistry, Seoul National University, Gwanak-gu, Seoul 151-742, Korea

**Keywords:** Cloned dog, De novo copy number variation, Mosaicism, Loss of heterozygosity, Somatic cell nuclear transfer

## Abstract

**Background:**

Somatic mosaicism of copy number variants (CNVs) in human body organs and de novo CNV event in monozygotic twins suggest that de novo CNVs can occur during mitotic recombination. These de novo CNV events are important for understanding genetic background of evolution and diverse phenotypes. In this study, we explored de novo CNV event in cloned dogs with identical genetic background.

**Results:**

We analyzed CNVs in seven cloned dogs using the nuclear donor genome as reference by array-CGH, and identified five de novo CNVs in two of the seven clones. Genomic qPCR, dye-swap array-CGH analysis and B-allele profile analysis were used for their validation. Two larger de novo CNVs (5.2 Mb and 338 Kb) on chromosomes X and 19 in clone-3 were consistently validated by all three experiments. The other three smaller CNVs (sized from 36.1 to76.4 Kb) on chromosomes 2, 15 and 32 in clone-3 and clone-6 were verified by at least one of the three validations. In addition to the de novo CNVs, we identified a 37 Mb-sized copy neutral de novo loss of heterozygosity event on chromosome 2 in clone-6.

**Conclusions:**

To our knowledge, this is the first report of de novo CNVs in the cloned dogs which were generated by somatic cell nuclear transfer technology. To study de novo genetic events in cloned animals can help understand formation mechanisms of genetic variants and their biological implications.

## Background

Together with single nucleotide polymorphisms (SNPs), DNA structural variations generally termed copy number variations (CNVs) are the major part of genomic variations in diverse animals including dogs [1,2]. CNVs are thought to contribute to phenotypic diversities including disease susceptibility and provide the important substrate for evolution [3,4]. Although it has been widely believed that all the cells in one individual are genetically identical, this dogma is changing due to our understanding of genomic variants. Indeed, recent studies have demonstrated the somatic mosaicism of CNVs in body organs [5,6], which suggests that de novo CNV events may commonly occur during mitotic recombination [7]. This possibility was clearly demonstrated by Bruder et al’s report identifying de novo somatic CNV events in monozygotic (MZ) twins [8]. They showed that the de novo post-twinning CNV frequency could be as high as 5% on a per-individual basis or 10% per twinning event. Also, Breckpot et al. reported CNVs in 1 out of 6 phenotypically discordant MZ twins [9]. These results suggest that CNV analysis in phenotypically discordant MZ twins can be useful to identify genetic loci associated with various traits. However, the de novo rate of somatic CNV mosaicism events is still largely unknown. For the case of cloned dogs, there has been no report about the de novo somatic CNV mosaicism event itself.

Dogs (*Canis familiaris*) have been used as working animals due to their superior sniffing ability to any other species or machine. For example, a cancer-sniffing dog can detect the existence of colorectal cancer from stool samples with 97% sensitivity and 99% specificity [10]. However, up to 70% of dogs that were bred from working dogs are not suitable for practical use because they lack trainability [11]. To reduce the tremendous cost, effort, and time to train one working dog, dog-cloning technology has been applied for the propagation of elite working dogs [12].

In this study, we aimed to identify de novo somatic CNV events which may exist in cloned dogs with identical genetic backgrounds like in human MZ twins using microarray-based comparative genomic hybridization (array-CGH). Array-CGH is one of the most popular and useful tools to analyze genome-wide CNV profiles and a number of studies have identified canine CNVs using this technique [13-17]. We validated the CNVs by diverse analyses such as genomic qPCR, dye-swap array-CGH, and B-allele profile analysis. We examined the genome-wide de novo somatic CNV events for the seven cloned dogs generated in our previous study [12] and identified five de novo CNV events.

## Results

### Validation of custom canine-array

**Figure 1:**
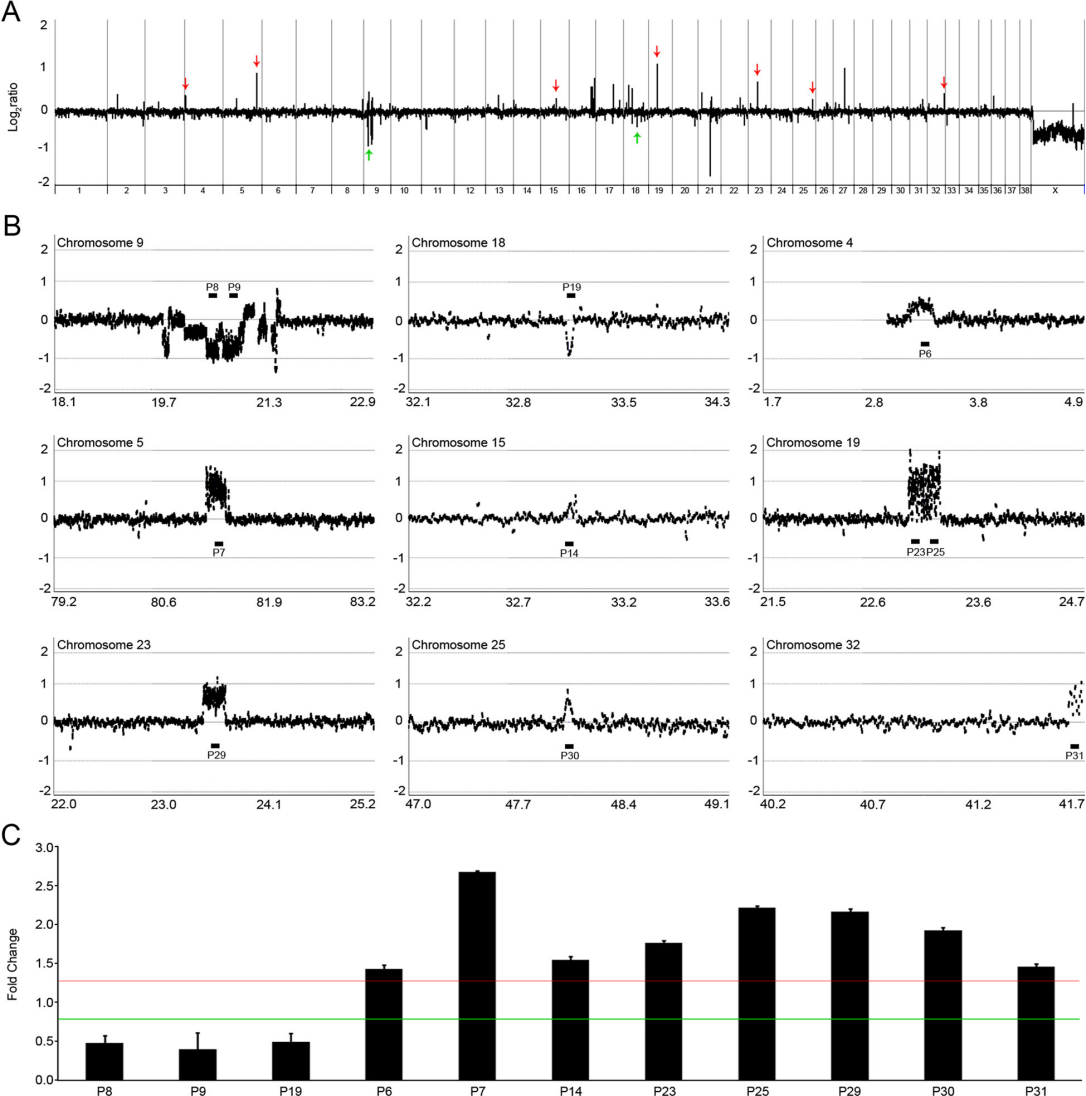
**Whole-genome plot of CNVs and qPCR validation. (A)** Whole-genome plot of CNVs identified between Labrador retriever and Boxer genomes. Nine CNV regions (7 CNV-Gs, red arrow; 2 CNV-Ls, green arrow) were randomly selected for qPCR validation. The X-axis represents individual chromosomes and the Y-axis represents signal intensity ratios (Labrador retriever/Boxer) on a log2 scale. **(B)** Log2ratio plot around the nine selected CNV regions. Black bars represent primer position for qPCR validation. The X-axis represents genomic position (Mb) and the Y-axis represents signal intensity ratios (Labrador retriever/Boxer) on log2 scale. **(C)** Three primer sets located in the CNV-L regions detected copy loss, and the other eight primer sets located in the CNV-G regions detected copy gain. The red and green line represent detection criterion for copy gain and copy loss, respectively. The Y-axis represents fold change based on the Boxer as the calibrator.

We designed a custom array containing an average probe spacing of 3.3 Kb across the whole canine genome for CNV screening. Before analyzing potential CNVs among the seven cloned dogs, we first validated whether this custom array platform could reliably detect CNVs between different canine breeds. For this, we screened CNVs between the nuclear donor, a Labrador retriever genome (male), and a Boxer genome as reference (female) using array-CGH, and verify the CNVs by genomic qPCR. Under our CNV detection criteria, we identified 72 CNVs between the Labrador retriever and Boxer across diverse chromosomes spanning 0.35% (8.6 Mb) of the canine genome. The average CNV size was 119 Kb (ranging from 11.1 to 450.6 Kb). Figure 1A illustrates the whole-genome plot of copy number gain- and loss-CNVs (CNV-G and CNV-L, respectively) identified with our array. Details of the 72 CNVs are available in Additional file 1: Table S1. When we compared the 72 CNVs with previously identified dog CNV regions [14-17], 90.3% (65 of 72 CNVs) identified in this study overlapped with the previously reported canine CNVs, while seven (9.7%) were novel. Statistical significance of enriched CNV overlap with known dog CNV data was evaluated by permutation test as described previously [18]. The overlap with known dog CNV data was significantly not a random event (*P* < 0.001). To validate our array platform and our CNV defining criteria through genomic qPCR, we randomly selected nine CNV loci (2 CNV-Ls, and 7 CNV-Gs) out of the 72 CNVs and designed eleven primer sets targeting the nine loci. All the eleven qPCRs in the nine CNV loci showed consistent copy number changes that matched the array results (Figures 1B, 1C). We also validated the seven novel CNVs. The average size of the novel CNVs was 103.5 Kb (ranging from 26.8 to 235.7 Kb) and all of them were consistently validated by genomic qPCR (Additional file 1: Figure S1). Since one of the 7 novel CNVs (on chromosome 4) was included in the nine validation targets, a total of 15 CNVs were qPCR validated in this study. All these data supported the reliability of our customized array platform and the subsequent analyses for identifying canine CNVs, therefore we applied this system to analyze cloned dog genomes.

### Association with segmental duplications and repetitive sequences

To understand the mechanism of CNV formation, we assessed the association of CNVs with segmental duplications (SDs) and repetitive sequences. When we compared the 72 CNVs with the CNV data from Nicholas et al’s report, which systematically analyzed SDs and associated CNVs in *canis familiaris *[15], 48 (67%) overlapped with the SDs. Total length of the overlapped CNVs was about 6 Mb (~70% of the total length of the 72 CNVs). This result was coherent with Nicholas et al’s previous report demonstrating that 70% of CNVs were associated with SDs [16]. Considering that SDs are often substrates of CNV formation via non allelelic homologous recombination (NAHR) [3], ours and previous results suggest that SD-associated NAHR might be the major mechanism of canine CNV generation. When we observed the genomic fraction (fraction of retroelements per kb) of long interspersed elements (LINE), short interspersed elements (SINE) and long terminal repeats (LTR) within (intraCNV) or in the vicinity of the CNVs (up to 10 Mb), LINE elements were relatively enriched the flanking regions (up to 10 kb away from CNVs), while relatively depleted LINEs/SINEs in intraCNV (Additional file 1: Figure S2). This result is coherent with the previous reports suggesting that repeated sequences may play a role in the formation of structural variation and genomic diversity [19,20].

### De novo CNVs identified in the cloned dogs

**Figure 2:**
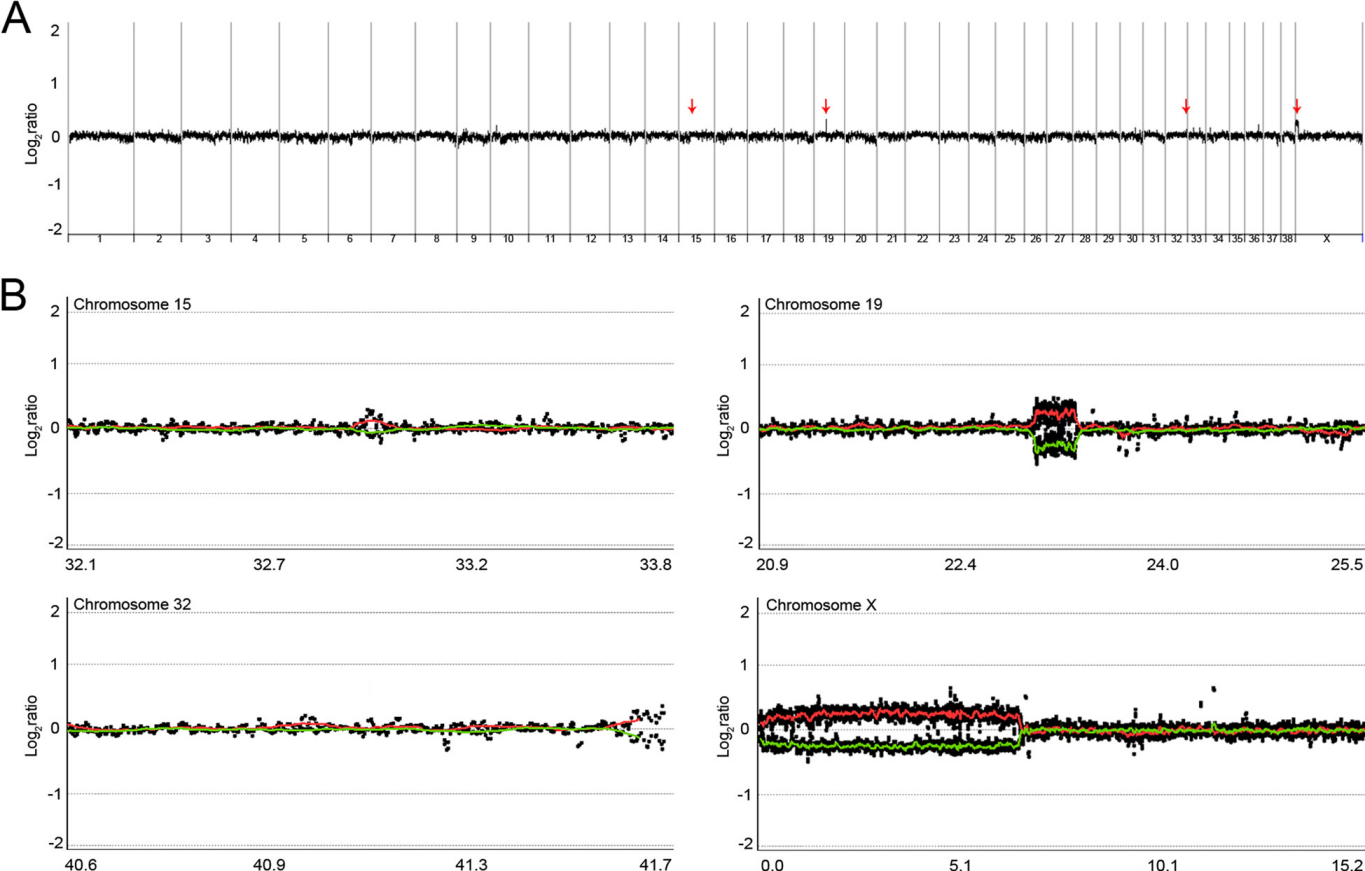
**Dye-swap analysis for the clone-3 genome. (A)** Genome-wide signal intensity plot from clone-3. Four CNV regions were identified in the clone-3 genome (red arrow). The X-axis represents individual chromosomes and the Y-axis represents signal intensity ratio (clone-3/donor) on a log2 scale. **(B)** Log2ratio plots around the four CNV regions in clone-3. All CNV-Gs identified in clone-3 (red line) show flipped signal intensity plots (green line) in the dye-swap hybridization. The X-axis represents genomic position (Mb), and the Y-axis represents signal intensity ratio on a log2 scale.

To verify the genetic homogeneity between the donor dog and clones, we first performed the microsatellite-based evaluation of the individual identity. Being consistent with our previous report [12], all seven cloned dogs showed identical microsatellite patterns with the donor at all nine microsatellite markers examined (Additional file 1: Table S2). We calculated the probability that the clone should have the same genotype as the donor under two different assumptions: assuming Hardy–Weinberg equilibrium, and assuming that both individuals are siblings as described previously [21,22]. The probabilities of exact allele matching at the nine microsatellite markers in a population of Labrador retriever (Hardy-Weinberg *P* = 0.00048 and Full siblings *P* = 0.01763) suggest that allele matching at all nine microsatellite loci is not a random event. We next performed genome-wide array-CGH-based CNV screening to identify de novo CNVs in cloned dogs. For this, genomic DNA from the nuclear donor dog was used as the universal reference for each array-CGH. Therefore, all the CNVs identified can be interpreted as de novo ones. Under the same CNV detection criteria as in the validation experiment, we identified five de novo CNVs in two of the seven cloned dogs ranging from 36 Kb to 5.2 Mb (Table 1). Among the five de novo CNVs, a 36 Kb-sized CNV on chromosome 2 (cloned dog-6) did not overlap with the previously reported canine CNVs, while the other four (cloned dog-3) overlapped with those reported (Table 1). The overlap with known dog CNV data was significantly not a random event (*P* < 0.001). Figure 2A is the genome-wide signal intensity plot (log2 scale) in cloned dog-3 (clone-3) as an example. To validate the de novo CNVs, we first carried out dye-swap array-CGH analysis for the clone-3 genome, but not for the clone-6 genome due to the lack of genomic DNA. Dye swap is a repeating hybridization on two-dye microarrays with the same samples but with swapped fluorescent labels. If there is true copy number change, the test/reference intensity ratio value must be inverted by the switching of the dyes. The concordant copy number changes detected in dye-swap analysis were considered to be true changes. As expected, all four CNVs identified in clone-3 consistently showed flipped signal intensity plots in the dye-swap hybridization (Figure 2B), supporting the accuracy of the CNVs identified by array-CGH.

**Table 1:** De novo CNVs in cloned dogs

**Clone**	**Chr**	**Start (bp)**	**End (bp)**	**Length (bp)**	**Event**	Genes^*^	**Validated by**^**†**^	**Reported CNV**^**§**^
Clone-3	15	32,983,895	33,026,044	42,150	CN Gain	-	Q, S	Yes
Clone-3	19	23,000,913	23,338,557	337,644	CN Gain	-	Q, S, B	Yes
Clone-3	32	41,654,990	41,731,424	76,435	CN Gain	-	Q, S	Yes
Clone-3	X	1,401,988	6,598,355	5,196,367	CN Gain	***ARSE, ARSH, OBP,**** GYG2, ARSD, ARSF, MXRA5, PRKX, PRKY, NLGN4T, NLGN4X, HDHD1, STS, PNPLA4, AKL1, TAF9, APOO, SHROOM2*	Q, S, B	Yes
Clone-6	2	71,652,481	71,688,546	36,066	CN Loss	*ZBTB8A, PCNAP1*	Q	No

*Bold text indicates reference genes in canine and regular text indicates reference genes in human.

†Q, genomic qPCR; S, dye-swap array-CGH analysis; B, B-allele profile analysis.

§Overlapped with the previously reported CNVs [14-17].

### B-allele profile of the de novo CNVs

**Figure 3:**
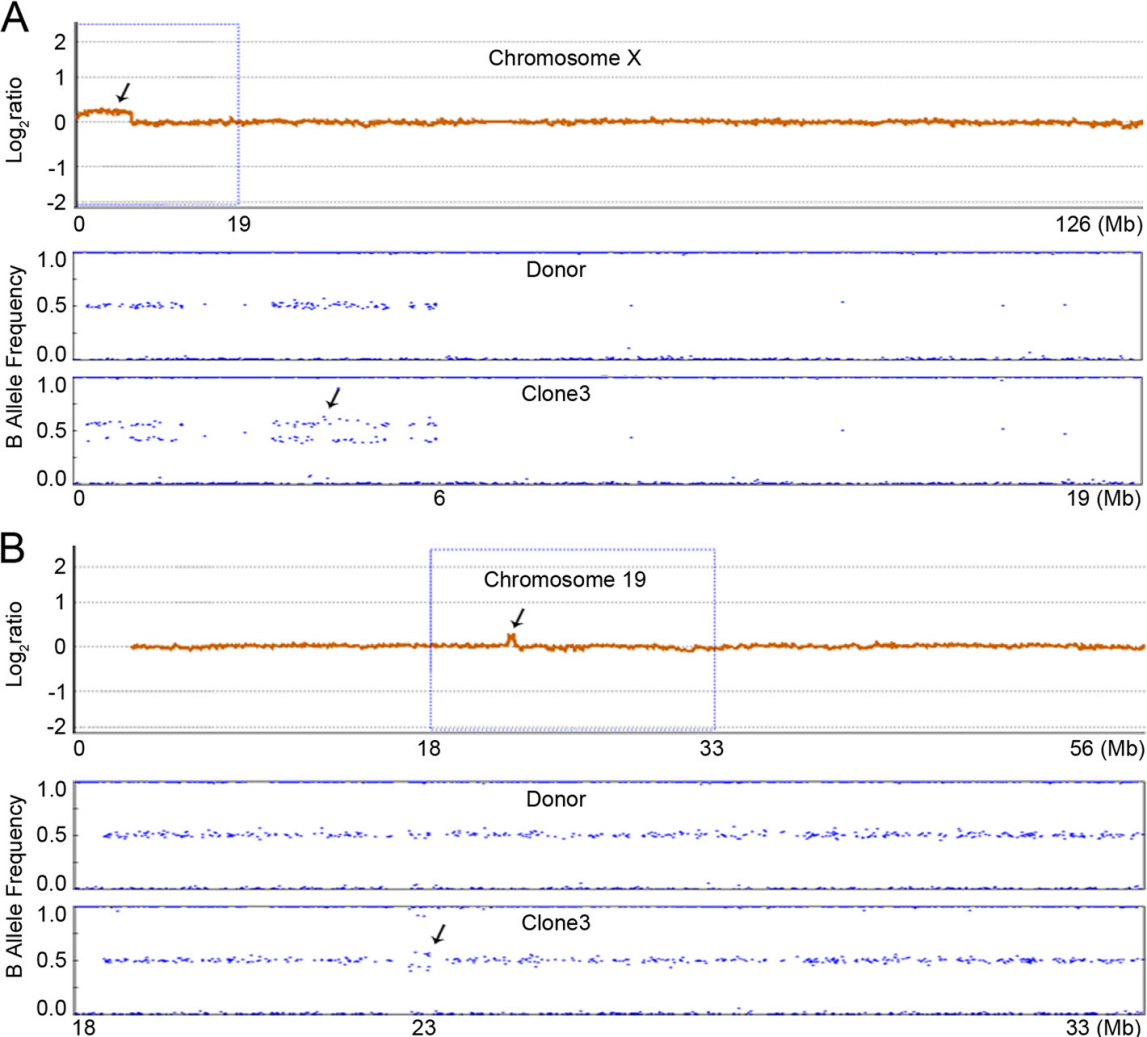
**Examples of allelic imbalance in clone-3. (A)** Upper panel, Log2ratio plot on chromosome X in clone 3. A 5.2-Mb copy number gain region on chromosome X was identified in clone-3 (black arrow). The X-axis represents genomic position (Mb) and the Y-axis represents signal intensity ratio on a log2 scale. Lower panel, B-allele plot of the proximal part of chromosome X (dashed box of upper panel). A heterozygous cluster was detected in chromosome X (black arrow), and this allelic imbalance has the same genomic position as the copy gain region. **(B)** Upper panel, Log2ratio plot on chromosome 19 in the clone-3. A 338-Kb copy number gain region on chromosome 19 was identified in clone-3 (black arrow). Lower panel, B-allele plot of the dashed box on chromosome 19. A heterozygous cluster was detected on chromosome 19 (black arrow), and this allelic imbalance has same position as the copy gain region.

In addition to the dye-swap analysis, we also performed whole-genome SNP genotyping for the genomes of the donor and cloned dogs using an Illumina CanineHD 170 K SNP array to obtain a more reliable interpretation of the de novo CNVs. A 5.2 Mb-sized region of copy number gain on chromosome X identified by array-CGH in clone-3 clearly showed two heterozygous clusters of SNPs in the B-allele plot, while no copy number gains or heterozygous clusters of SNPs were detected in the donor genome (Figure 3A). The 338 Kb-sized copy number gain on chromosome 19 of clone-3 also showed two heterozygous clusters of SNPs in the B-allele plot that were not detected in the donor genome (Figure 3B). The other three CNVs on chromosome 2, 15 and 32 were too small to be interpreted by B-allele pattern (Additional file 1: Figure S3).

**Figure 4:**
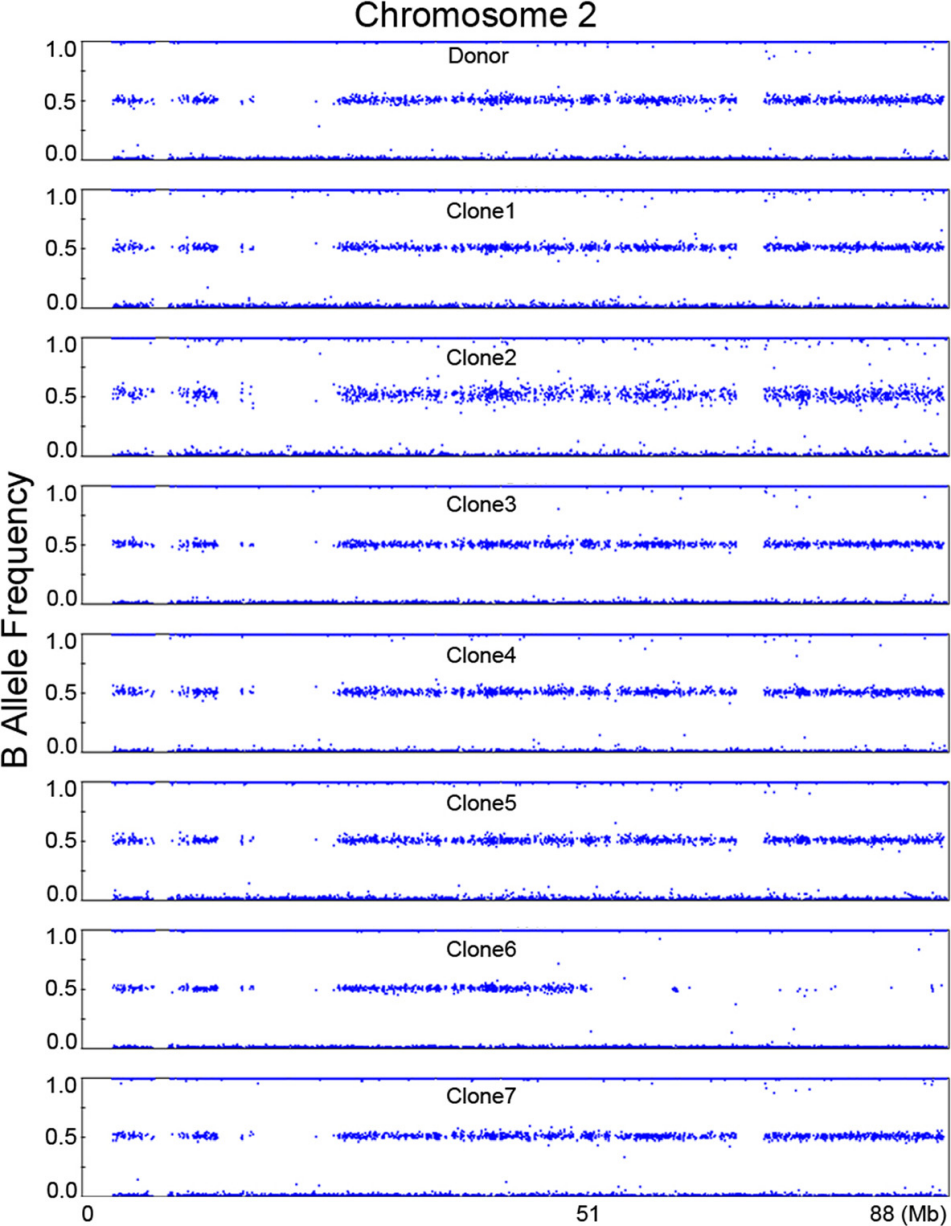
**Example of the LOH event in clone-6.** A 37-Mb de novo LOH event was identified on the distal end of chromosome 2 in clone-6 that was not detected in the donor or other clones. The X-axis represents genomic position (Mb), and the Y-axis represents B-allele intensity.

In addition to the de novo CNVs, we identified a copy neutral de novo loss of heterozygosity (LOH) event on chromosome 2 by the B-allele pattern analysis. This 37 Mb-sized LOH was identified in the distal end of chromosome 2 in clone-6, but not in the nuclear donor nor in the other cloned dogs (Figure 4).

### Genomic qPCR validation of the de novo CNVs

**Figure 5:**
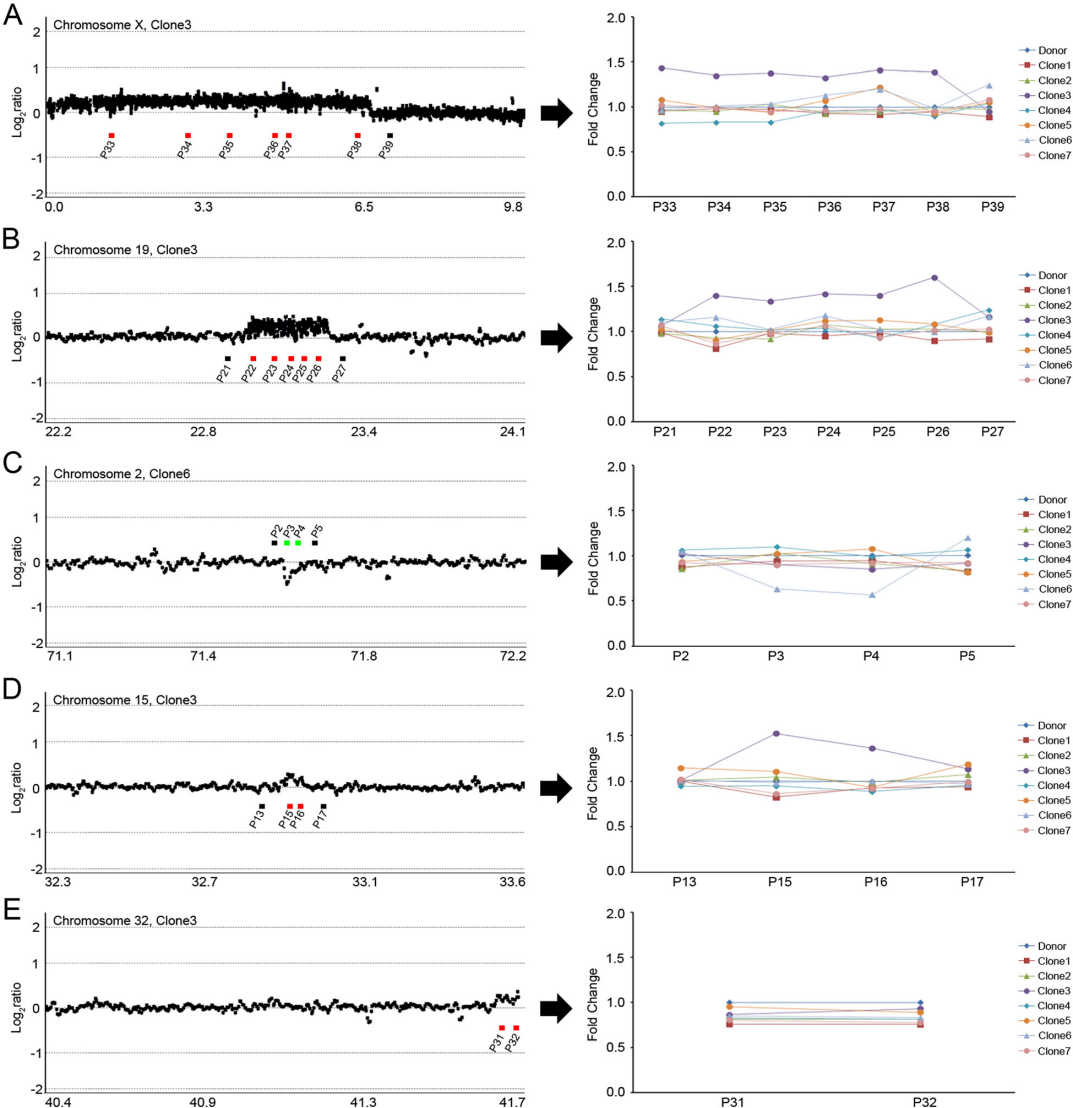
**Multiple-point genomic qPCR validation.** Left, Log2ratio plot on chromosome X **(A)**, chromosome 19 **(B)**, chromosome 15 **(D)**, chromosome 32 **(E)** in clone 3 and chromosome 2 **(C)** in clone-6. Red and green bars represent primer position within the CNV-Gs and CNV-Ls, respectively. Black bars represent primer position outside the expected CNV breakpoint. The X-axis represents genomic position (Mb) and the Y-axis represents signal intensity ratio on a log2 scale. Right, multiple-point genomic qPCR validation. The Y-axis represents fold-change based on the nuclear donor as calibrator.

To validate the existence and boundaries of the CNVs, we performed genomic qPCR for multiple points within and outside the expected CNV breakpoints. For this, we designed two to seven target specific primer pairs for each CNV (Additional file 1: Table S3). Through multiple-point genomic qPCR, four of the five CNVs were consistently validated, while the other one relatively smaller sized CNV was not validated (Figure 5). In the 5.2 Mb-sized region of copy number gain on chromosome X and the 338 Kb-sized gain on chromosome 19 in clone-3, all the qPCRs within the CNV regions consistently showed copy number gains and the qPCRs outside CNV region showed diploid copy numbers, which confirmed the accuracy of our array-CGH analysis in defining boundaries (Figures 5A, 5B). Likewise, all the other cloned dogs containing no CNVs were diploid at all qPCR points. Of the relatively smaller-sized CNVs, a 36 Kb copy number loss on chromosome 2 in clone-6 and 42 Kb gain on chromosome 15 in clone-3 were successfully validated and their boundaries were estimated by qPCR (Figure 5C, 5D). However, the 76 Kb-sized CNV-G on chromosome 32 in clone-3 was not validated by genomic qPCR (Figure 5E).

## Discussion

In this study, we aimed to identify de novo post-cloning CNV events which may exist in cloned dogs like in human MZ twins. For this purpose, we designed a 720 K custom array by merging the NimbleGen 385 K dog array as a backbone and 335 K probes for known canine CNV regions [14]. Using this platform, we identified five de novo CNVs in two of the seven cloned dogs from the same nuclear donor. Our findings support the occurrence of de novo post-cloning CNV events in mammals including dogs. Although de novo CNV events in phenotypically concordant MZ twins have been reported in humans [8], to the best of our knowledge, this is the first report of de novo CNVs in cloned animals including dogs generated by SCNT technology.

As mentioned above, the de novo CNVs were unevenly identified across the dog clones (2/7 clones); four CNVs were identified in one clone (clone-3) and one was in another clone (clone-6). Our result is similar with the previous reports observing the CNVs in human MZ twins. Breckpot et al. identified CNVs in 1 out of 6 phenotypically discordant MZ twins [9]. In Ehli et al’s report with 50 concordant and discordant MZ twins, 18 de novo post-twining CNVs were detected in 14 out of 45 twin pairs [23]. However, we cannot rule out the possibility of existence of more de novo CNVs because the array platform and defining algorithm we used in this study cannot be perfect. Also, it is possible that some genetic events may have happened in the clone-3 harboring 4 CNVs during its development or SCNT procedure. Further CNV studies with a larger number of cloned dogs will be required to understand this phenomenon.

When we screened CNVs between the donor Labrador retriever and a Boxer to validate the performance of our custom array, the number of CNVs was largely similar to the report by Nicholas et al. that compared CNVs using a 2.1 M array between nine breeds of modern domestic dogs including Labrador retriever [16]. Most of the CNVs (90.3%) identified in our validation experiment overlapped with the previously reported CNVs [14-17], and the overlap was significantly not random event (*P* < 0.001). All the nine randomly selected CNV loci and seven novel CNVs identified by array-CGH showed consistent copy number changes that were validated by qPCR. These results suggest the reliability of our custom array and CNV defining procedure. Although exploring the novel canine CNVs is not our aim in this study, this result suggests that a substantial number of canine CNVs that have yet to be identified.

To validate the five de novo CNVs in the cloned dogs, we performed three different verification experiments; genomic qPCR, dye-swap array-CGH analysis and B-allele profile analysis. Two larger de novo CNVs (5.2 Mb and 338 Kb) on chromosomes X and 19 in clone-3 were consistently validated by all three experiments. The other three CNVs on chromosomes 2, 15, and 32 (36.1 - 76.4 Kb) seem to be too small to be interpreted by B-allele pattern (Additional file 1: Figure S3). According to the technical note about CNV analysis algorithm for Illumina HumanHap240S array, which has a higher SNP density than the array used in this study (170 K), the minimum recommended window size for LOH score algorithm is 360 Kb [24]. Of the three smaller-sized CNVs, two (a CNV on chromosome 2 in clone-6 and another one on chromosome 15 in clone-3) were verified by qPCR, but the CNV on the distal end of chromosome 32 was not verified. However, the CNV region on chromosome 32 was validated in dye-swap analysis, which suggests that the 76 Kb-sized CNV region on chromosome 32 might be real.

There are several genes in the 5.2 Mb-sized copy number gain CNV locus including the neuroligin 4 X-linked (*NLGN4X*), steroid sulfatase isozyme S (*STS*), and odorant-binding protein (*OBP*) genes. In human, genetic variants of the *NLGN4X* gene have been reported to be associated with autism spectrum disorder in Japanese and Han Chinese populations [25-27]. A CNV of the *STS* gene (duplication) has also been reported to be associated with intellectual disability in human [28]. OBP is small soluble carrier protein that belongs to the family of lipocalins and that may be involved in the perception of pheromones [29]. However, we did not observe any differences regarding the phenotypes related to these genes in the cloned dogs harboring the de novo CNVs in this study.

In addition to the de novo CNVs, we also detected a copy neutral de novo LOH event on chromosome 2. This 37 Mb-sized LOH event occurred in a cloned dog but was not present in the nuclear donor dog, indicating that the uniparental disomy (UPD) arose from a mitotic event in the cloned dog. It is well known that UPD is a genetic event associated with human diseases and mosaicism of aneuploidy can be associated with UPD [30]. Our results suggest that SCNT could result in UPD-related phenotypic differences among clones.

There are several limitations in this study. First, since the sample size of this study is very small, the scope of our analysis could not go beyond describing de novo CNVs. Second, even though we identified de novo CNVs in cloned dogs, we could not analyze any association between the CNVs and phenotypic characteristics, because there have been no phenotypic differences among them so far. To overcome both limitations and draw biologically meaningful conclusions, we need to clone more dogs and to carefully observe the phenotypes of them, and apply more informative technologies such as whole genome sequencing.

## Conclusions

In conclusion, we identified de novo CNV and LOH events in cloned dogs generated from one nuclear donor. To the best of our knowledge, this is the first report of de novo CNVs in cloned animals including dogs generated by SCNT technology. Since cloned animals share almost identical genetic background like MZ twins, to study their de novo genetic events can help understand formation mechanisms of genetic variants and their biological implications. Also, our results can be a useful tool to study the genetic basis of diverse disease/phenotype traits in dogs.

## Methods

### Source of dog DNA samples

Seven cloned Labrador retriever dogs named clone-1 to -7 were generated by somatic cell nuclear transfer (SCNT) using fibroblasts derived from a seven-year-old Labrador retriever, and the genetic homogeneity was validated by microsatellite analysis using nine markers (PEZ01, PEZ02, PEZ03, PEZ06, PEZ13, PEZ17, FH2079, FH2054, FH2010) in the previous study [12]. In order to extract genomic DNA samples, blood was collected from the seven cloned dogs at three years of age and from the donor dog at ten years of age. Approximately 5 ml of blood was collected into anticoagulant tubes containing EDTA. Genomic DNA was extracted from whole-blood using a DNeasy Blood & Tissue Kit (Qiagen, Hilden, Germany) and quantified by NanoDrop spectrophotometer. This study was performed with the approval of animal ethics committee of Seoul National University.

### Whole-genome array-CGH experiment

We used a NimbleGen custom 720K canine whole-genome array platform (Roche NimbleGen, Penzberg, Germany), designed from the UCSC dog genome build of May 2005 (Broad/canFam2). This array is comprised of 385,000 probes evenly distributed throughout the unique sequence of the genome as a backbone and an additional ~330,000 probes which were designed based on previously reported dog CNV regions [14]. The average probe spacing for our custom array platform was ~3.3 Kb. The array-CGH experiments were performed according to the manufacturer’s instructions. In brief, 1 μg genomic DNA from each cloned dog was labeled with Cy3-dCTP. The reference DNA was labeled with Cy5-dCTP. Each dye-labeled DNA was purified and mixed with hybridization reagents, applied on the array and incubated for 48 hours at 42°C in a MAUI hybridization machine (BioMicro, Salt Lake City, UT). After washing the slides, arrays were scanned using a GenePix 4000B scanner (Axon Instruments, Sunnyvale, CA). Image separation, signal intensity extraction, and normalization were performed using the NimbleScan software version 2.5 (Roche NimbleGen).

### Detection of CNVs

The Rank-Segmentation CNV calling algorithm in NEXUS software v3.1 (BioDiscovery Inc., El Segundo, CA) was used to define CNVs of each sample. The parameters for defining CNVs were as follows: significance threshold = 1.0E-6; maximum contiguous probe spacing (Kbp) = 1000 Kb; minimum number of contiguous probes per CNV segment = 5; threshold of signal intensity ratio > 0.2 on the log2 scale for gains and < -0.2 on the log2 scale for losses.

### Whole-genome SNP genotyping

Genome-wide SNP genotyping was conducted using the CanineHD SNP array, which contains 173,662 SNP markers (Illumina, Inc., San Diego, CA) according to manufacturer’s instructions. The average probe spacing is 13 Kb. The raw data consisted of the signal intensity (log R ratio: LRR) and allelic intensity (B allele frequency) that were obtained using GenomeStudio software (Illumina, Inc.). Based on the B allele frequency profile, we defined the CNVs according to the concept of integrating SNP and CNV genotyping [3]. Samples that did not have a standard deviation (SD) of LRR < 0.24 were excluded for quality control. The SNPRank Segmentation algorithm in NEXUS software was used to define allele imbalance of each sample.

### Genomic quantitative PCR analysis

Array-CGH results were experimentally validated by genomic qPCR in randomly selected CNV loci for boxer and all CNV loci for cloned dogs. As a diploid internal control, a genomic region on chr7:18946362 that showed no genomic alteration in the array-CGH data was used. Details including primer information for targets and the diploid control locus are available in Additional file 1: Table S3. Genomic qPCR was performed using the ViiA7™ real-time PCR system (Life Technologies, Carlsbad, CA) as describe elsewhere [31]. In brief, a 10 μl of reaction mixture contained 10 ng of genomic DNA, THUNDERBIRD™ SYBR qPCR Mix (TOYOBO, Osaka, Japan), 1× ROX, and 6 pmole of each primer. Thermal cycling conditions consisted of one cycle of 1 min at 95°C followed by 40 cycles of 5 sec at 95°C, 10 sec at 61°C, and 20 sec at 72°C. All qPCR experiments were triplicate and the copy number of each target was defined as 2^-ΔΔCT^, where ΔC_t_ is the difference in threshold cycles of the sample in question normalized against the reference region and expressed relative to the value obtained by calibrator DNA (individual/calibrator) as describe elsewhere [32]. DNA from the nuclear donor dog was used as calibrator for all qPCR experiment.

### Probability of identity

Probability of identity (PI) for multilocus microsatellite data was computed using GENECAP program [33] under the two different assumptions of Hardy-Weinberg equilibrium and the sibling PI as described previously [21,22]. Where, PI indicates the probability of two individuals within the population sharing the same genotype. In GENECAP, multilocus PI values were computed by mutiplying all locus-specific PIs under the assumption that each locus is independent.

### Significance of enriched CNV overlap with known dog CNV data

Statistical significance of enriched CNV overlap with known dog CNV data was evaluated by permutation test as described previously [18]. In this test, original CNV overlap with database was calculated based on >1 bp overlap measure. In each iteration, CNVs were randomly reshuffled while maintaining their sizes and numbers within each chromosome. Based on these permuted CNVs, we calculated the overlap with the same database. After repeating this process 1000 times, we calculated P-value as the fraction of tests where the number of overlaps was greater than the original unpermuted CNV overlap.

### Statistical analyses

Statistical analyses were performed using SPSS version 18.0 (SPSS Inc., Chicago, IL). *P* values < 0.05 were considered significant.

### Availability of supporting data

The array-CGH data (NimbleGen custom 720 K canine whole-genome array) and the SNP genotyping data (Illumina CanineHD SNP array) from this study are submitted to the NCBI Gene Expression omnibus (http://www.ncbi.nlm.nih.gov/geo) under accession no GSE49092 and GSE49123, respectively. The following supporting data are available with the online version of this paper.

## Abbreviations

CNV: Copy number variant; SCNT: Somatic cell nuclear transfer; Array-CGH: Microarray-comparative genomic hybridization; qPCR: Quantitative polymerase chain reaction; SNP: Single nucleotide polymorphism; MZ: Monozygotic; LOH: Loss of heterozygosity; Kb: Kilo base pair; LRR: Log R ratio; SD: Standard deviation.

## Competing interests

The authors declare that they have no competing interests.

## Authors’ contributions

YJC, BCL, JSK made conception and study design, SHJ carried out array-CGH, CNV detection, SNP genotyping and genomic qPCR validation, SHY, and TMK carried out CNV determination and data interpretation of molecular genetics studies, HJO, JEP, MJK, and GAK carried out sample collection and genomic qPCR validation analyses, YJC and SHJ analyzed the whole data, YJC, BCL, SHJ and SHY wrote the paper. All authors read and approved the final manuscript.

## Supplementary Material

Additional file 1 **Table S1.** CNVs between Labrador retriever and Boxer genome. **Table S2.** Microsatellite analysis of the donor cell and cloned dogs using nine canine specific microsatellite markers. **Table S3.** Sequence information of the primers for genomic qPCR validation. **Figure S1.** Novel CNVs identified between a Labrador retriever and a Boxer, and qPCR validation. **Figure S2.** Genomic fraction of the repetitive sequence elements. **Figure S3.** B allele profiles around the three small-sized de novo CNVs. Click here for file
