# Supplementary material for: De novo copy number variations in cloned dogs from the same nuclear donor
Source: BMC Genomics. 2013 Dec 9;14:863. doi: 10.1186/1471-2164-14-863 (PMC3878922; doi:10.1186/1471-2164-14-863)
Supplement: Additional file 1 — Table S1. CNVs between Labrador retriever and Boxer genome. Table S2. Microsatellite analysis of the donor cell and cloned dogs using nine canine specific microsatellite markers. Table S3. Sequence information of the primers for genomic qPCR validation. Figure S1. Novel CNVs identified between a Labrador retriever and a Boxer, and qPCR validation. Figure S2. Genomic fraction of the repetitive sequence elements. Figure S3. B allele profiles around the three small-sized de novo CNVs. [file 1471-2164-14-863-S1.doc]

**Supplementary Table S1**. CNVs between Labrador retriever and Boxer genome

| **Chr** | **Start (bp)** | **End (bp)** | **Length (bp)** | **Event*** | **Reported CNV^**^** | **Genes§** |
| --- | --- | --- | --- | --- | --- | --- |
| 2 | 26,023,564 | 26,089,330 | 65,766 | Loss | Yes | ***LOC478000****,* ***LOC478001****, PHYH* |
| 2 | 86,823,708 | 86,971,711 | 148,004 | Loss | Yes | *VPS13D* |
| 4 | 3,246,235 | 3,481,886 | 235,652 | Loss | No | *SLMO2* |
| 5 | 34,076,840 | 34,185,868 | 109,028 | Loss | Yes |  |
| 5 | 81,155,598 | 81,174,544 | 18,947 | Loss | Yes |  |
| 5 | 81,174,544 | 81,406,432 | 231,889 | Loss | Yes |  |
| 5 | 81,438,815 | 81,451,764 | 12,950 | Loss | Yes |  |
| 6 | 19,839,399 | 19,907,659 | 68,261 | Gain | Yes | ***ITGAD****, ZSCAN2, ZNF691, ZNF239, ZNF16, ZNF629, ZNF197, ZNF229, ZNF225, ZNF221, ZNF30, ZNF12, ZNF226, ZSCAN20, ZNF436, ZNF329, ZNF585A, ZNF224, ZNF616, ZNF48, ITGAM, ITGAX,* |
| 8 | 76,471,254 | 76,557,900 | 86,647 | Loss | Yes |  |
| 8 | 76,842,192 | 76,882,003 | 39,812 | Loss | Yes |  |
| 9 | 10,427,294 | 10,513,294 | 86,001 | Gain | Yes |  |
| 9 | 11,618,798 | 11,740,208 | 121,410 | Gain | Yes |  |
| 9 | 11,740,208 | 11,851,646 | 111,439 | Loss | Yes |  |
| 9 | 13,462,313 | 13,506,673 | 44,360 | Loss | Yes | *PLEKHM1, PLEKHM1P, ARHGAP27* |
| 9 | 19,785,216 | 19,874,985 | 89,770 | Gain | Yes |  |
| 9 | 20,111,420 | 20,446,507 | 335,088 | Gain | Yes | *CASC17* |
| 9 | 20,446,507 | 20,627,634 | 181,128 | Gain | Yes | *CASC17* |
| 9 | 20,627,634 | 20,694,280 | 66,647 | Gain | Yes |  |
| 9 | 20,694,280 | 20,935,074 | 240,794 | Gain | Yes |  |
| 9 | 20,935,074 | 21,009,886 | 74,813 | Gain | Yes |  |
| 9 | 21,020,830 | 21,198,522 | 177,692 | Loss | Yes | *VPS13D* |
| 9 | 21,198,522 | 21,274,612 | 76,091 | Gain | Yes | *MAP3K14-AS1* |
| 9 | 21,300,781 | 21,313,768 | 12,988 | Gain | Yes | *MAP3K14-AS1, LRRC37B, LRRC37A6P, LRRC37A3, LRRC37A, LRRC37A2, LRRC37A11P* |
| 9 | 21,340,889 | 21,505,846 | 164,958 | Gain | Yes | *MAP3K14-AS1, LRRC37B, LRRC37A6P, LRRC37A11P, ZC3HC1, ARHGAP27* |
| 9 | 21,512,886 | 21,562,024 | 49,139 | Loss | Yes | *MAP3K14-AS1* |
| 9 | 63,910,095 | 63,972,864 | 62,769 | Loss | No | *TTLL11* |
| 10 | 5,498,324 | 5,525,140 | 26,817 | Loss | No |  |
| 11 | 12,165,554 | 12,616,105 | 450,552 | Gain | Yes | *FAM170A* |
| 11 | 13,808,458 | 13,990,419 | 181,962 | Gain | Yes |  |
| 11 | 14,065,859 | 14,423,826 | 357,968 | Gain | Yes |  |
| 13 | 7,987,006 | 8,040,498 | 53,493 | Loss | Yes | *RIMS2, RIMS1* |
| 13 | 32,014,995 | 32,125,614 | 110,620 | Loss | No | *KCNQ3, KCNQ2* |
| 13 | 44,476,157 | 44,515,880 | 39,723 | Loss | Yes |  |
| 15 | 32,980,351 | 33,026,044 | 45,694 | Loss | Yes |  |
| 15 | 38,717,467 | 38,756,482 | 39,016 | Loss | Yes | *CCDC38, MDH2* |
| 16 | 53,617,206 | 53,798,615 | 181,410 | Loss | Yes |  |
| 16 | 57,003,933 | 57,122,436 | 118,504 | Loss | No | *FAM90A10P, USP17L8, USP17L1P, USP17LL4, USP17L15, USP17L2, USP17L10, USP17L5, USP17L19, USP17L3, USP17L11, USP17L17, USP17L12, USP17L18, USP17L22, USP17L13, USP17L21, USP17L20, USP17L7, USP17L4, USP17L29, USP17L26, USP17L24, USP17L25, USP17L27, USP17L6P, USP17L28, USP17L9P, USP17L30* |
| 16 | 57,867,557 | 57,916,743 | 49,187 | Loss | Yes |  |
| 16 | 61,896,791 | 62,319,097 | 422,307 | Loss | Yes | ***CBD103****,* ***SPAG11E****,* ***SPAG11B****,* ***DEFB105A****, DEFB103A, DEFB103B, SPAG11A, DEFB104B, DEFB104A, DEFB106B, DEFB106A, DEFB105B, DEFB107B, DEFB107A, FAM90A2P, USP17L29, USP17L8, USP17L26, USP17L24, USP17L21, USP17L10, USP17L25, USP17L1P, USP17L5, USP17L15, USP17L6P, USP17L30, USP17L27, USP17L19, USP17L2, USP17L28, USP17L9P, USP17L3, USP17L17, USP17L11, USP17L12, USP17L18, USP17L22, USP17L13, USP17L20, USP17L7, USP17L4, UBE2F, RALB, RBM4, RBM4B, OTUD6B* |
| 16 | 62,319,097 | 62,330,201 | 11,104 | Loss | Yes | *DEFB107B, DEFB107A, FAM90A2P* |
| 16 | 62,330,201 | 62,570,175 | 239,975 | Loss | Yes | *FAM90A2P, FAM90A25P* |
| 17 | 24,437,738 | 24,548,926 | 111,189 | Gain | Yes | *GCKR, MRPL21, C2orf16* |
| 17 | 42,382,735 | 42,493,380 | 110,646 | Gain | Yes | *SFTPB* |
| 17 | 44,631,192 | 44,820,798 | 189,606 | Loss | Yes |  |
| 17 | 59,753,592 | 59,896,503 | 142,912 | Loss | Yes | *HMGCS2, REG4, ADAM30, PHGDH* |
| 17 | 64,670,204 | 64,709,812 | 39,609 | Loss | Yes | *LCE1C, LCE1D, LCE1A, LCE4A, LCE3B, LCE1C, LCE2B, LCE2C, LCE2D, LCE2A, LCE3D, LCE1B, LCE3C, LCE1E, LCE3A, LCE3E, LCE1F, KRTAP17-1* |
| 18 | 14,310,085 | 14,415,570 | 105,486 | Loss | Yes | *TARP* |
| 18 | 21,348,826 | 21,412,450 | 63,624 | Gain | Yes | *MAGI2* |
| 18 | 21,437,905 | 21,570,637 | 132,732 | Loss | Yes | *MAGI2* |
| 18 | 21,689,351 | 21,832,888 | 143,538 | Loss | Yes | *MAGI2* |
| 18 | 33,231,283 | 33,280,654 | 49,372 | Gain | Yes | *RPS27L* |
| 18 | 47,680,022 | 47,738,447 | 58,426 | Loss | No | *CD82* |
| 18 | 52,074,590 | 52,177,690 | 103,101 | Gain | Yes | *IGHMBP2, MAP2K1, MRPL21, CPT1A* |
| 18 | 57,295,517 | 57,346,153 | 50,636 | Gain | Yes | *SCGB2A1* |
| 19 | 23,020,008 | 23,331,919 | 311,911 | Loss | Yes |  |
| 21 | 10,527,115 | 10,966,770 | 439,656 | Loss | Yes | *MTNR1B, FAT3, RPS24* |
| 21 | 29,734,196 | 29,763,184 | 28,989 | Gain | Yes |  |
| 21 | 32,290,069 | 32,361,888 | 71,820 | Gain | Yes | ***COR56A6****, OR1B1, OR56A4* |
| 21 | 33,672,669 | 33,843,016 | 170,348 | Loss | Yes | ***OR2D3****, OR10A4, OR1B1, OR6S1, NPM2, MSI2, OR2D2* |
| 21 | 43,816,192 | 43,899,436 | 83,244 | Gain | Yes | ***SAA1****, C15orf40, SAAL1, SAA3P, SAA4, SAA2* |
| 22 | 46,315,901 | 46,427,949 | 112,048 | Loss | No | *GPC5* |
| 22 | 56,693,460 | 56,734,044 | 40,585 | Gain | Yes |  |
| 23 | 23,551,346 | 23,772,744 | 221,399 | Loss | Yes |  |
| 25 | 48,053,573 | 48,112,873 | 59,301 | Loss | Yes | *HJURP, UGT1A9, UGT1A8, UGT1A7, UGT1A10, UGT1A6* |
| 26 | 30,496,807 | 30,604,134 | 107,327 | Loss | Yes |  |
| 27 | 6,530,674 | 6,591,545 | 60,872 | Loss | Yes | ***CELA1****, GALNT6, GALNT3, BIN2* |
| 27 | 28,737,178 | 28,756,409 | 19,232 | Loss | Yes | *MAZ, SLC7A3* |
| 27 | 28,890,686 | 28,922,661 | 31,976 | Loss | Yes | *PYROXD1, MAZ, SLC7A3* |
| 31 | 33,443,182 | 33,497,535 | 54,354 | Loss | Yes |  |
| 32 | 41,649,592 | 41,731,424 | 81,833 | Loss | Yes |  |
| 33 | 5,248,223 | 5,330,514 | 82,292 | Gain | Yes | *NDRG4* |
| 36 | 7,019,571 | 7,079,295 | 59,724 | Loss | Yes | *UPP2* |

* CNV event is defined by a Boxer genome as reference.

** Overlapped with the previously reported CNVs [14-17]

§ Bold text indicates reference genes in canine and regular text indicates reference genes in human

**Supplementary Table S2**. Microsatellite analysis of the donor cell and cloned dogs using nine canine specific microsatellite markers

| Marker | PEZ 01 | PEZ 03 | PEZ 02 | PEZ 06 | PEZ 13 | PEZ 17 | FH 2079 | FH 2054 | FH 2010 |
| --- | --- | --- | --- | --- | --- | --- | --- | --- | --- |
| Donor Cell | 106/122 | 118/140 | 127/131 | 179/179 | 221/233 | 207/215 | 150/154 | 269/277 | 228/232 |
| Clone 1 | 106/122 | 118/140 | 127/131 | 179/179 | 221/221 | 207/215 | 150/154 | 269/277 | 228/232 |
| Clone 2 | 106/122 | 118/140 | 127/131 | 179/179 | 221/221 | 207/215 | 150/154 | 269/277 | 228/232 |
| Clone 3 | 106/122 | 118/140 | 127/131 | 179/179 | 221/221 | 207/215 | 150/154 | 269/277 | 228/232 |
| Clone 4 | 106/122 | 118/140 | 127/131 | 179/179 | 221/221 | 207/215 | 150/154 | 269/277 | 228/232 |
| Clone 5 | 106/122 | 118/140 | 127/131 | 179/179 | 221/221 | 207/215 | 150/154 | 269/277 | 228/232 |
| Clone 6 | 106/122 | 118/140 | 127/131 | 179/179 | 221/221 | 207/215 | 150/154 | 269/277 | 228/232 |
| Clone 7 | 106/122 | 118/140 | 127/131 | 179/179 | 221/221 | 207/215 | 150/154 | 269/277 | 228/232 |

Alleles are named for the total length of the segment ampliﬁed.

This table was also represented in our preciously report [Oh et al., 2009, Theriogenology].

**Supplementary Table S3**. Sequence information of the primers for genomic qPCR validation

| **Primer ID** | **Chr** | **Position** | **Amplicon Size** | **Forward** | **Reverse** | **Event Type** |
| --- | --- | --- | --- | --- | --- | --- |
| Primer01 | 7 | 18,946,362-18,946,470 | 109 | ACAAAGAGGTGTGGGAGAAGCAGT | ACTGTGCAGAGCACTCCAGAAGAA | Diploid control |
| Primer02 | 2 | 71,649,025-71,649,139 | 115 | AGGCAGCATGACTTCTTCAGGGAT | TCAGGGTCTGGTGCCTACATACTT | Loss |
| Primer03 | 2 | 71,667,395-71,667,561 | 167 | AGGCACCACAAAGCTACTCTGGAT | TGTGAACATTCTCTCAGCCAGCCT | Loss |
| Primer04 | 2 | 71,672,828-71,672,960 | 133 | TGTGGTAGCAGTGGTGGAGTCAAA | TCTCATCAATGAAGCCTGCTGGGA | Loss |
| Primer05 | 2 | 71,690,827-71,690,968 | 142 | TACAGAGCAGCTGCATCCTTTCCT | TATGTGGGTAGGTGCCACCAATGA | Loss |
| Primer06 | 4 | 3351248-3351327 | 80 | TGCACCCAGCAAGTTGTCTTTGTC | AGGGCACAATGAGGAAGACTCCAA | Gain |
| Primer07 | 5 | 81,164,684-81,164,812 | 129 | TCTGGTGTGACTGCATGGATGGAT | AAGAAGGAAGGAAAGGTGCAGGGA | Gain |
| Primer08 | 9 | 20,557,654-20,557,834 | 181 | TTGGTGCAGCTTTGACCAATGACC | TAACCAAGCCTGCCTTCGTACTGT | Loss |
| Primer09 | 9 | 20,733,503-20,733,690 | 188 | TACGTGATTTGTGGCTTTGGTGGC | CAAAGTGCAACGTGCCGTTCCTAA | Loss |
| Primer10 | 9 | 63,935,643-63,935,742 | 100 | TGGGATGGGCAGTCTCTATAA | GCCACTAAGAGCCCAGAATAC | Gain |
| Primer11 | 10 | 5,509,100-5,509,223 | 124 | CAGAGAGGAGAAGGGTTGATTAG | GGAGGGAAGATGAGAAGAAGAAG | Gain |
| Primer12 | 13 | 32,043,618-32,043,720 | 103 | GATCCAGTGATGCTGGGAATAG | AGTCCTTTGTCTCTGGGTTTG | Gain |
| Primer13 | 15 | 32,979,555-32,979,744 | 190 | ACAGAAATCCTGCTGTCCACCTCA | TTGCTTCAGTTAAAGTGCCTGCCG | Gain |
| Primer14 | 15 | 32,990,154-32,990,244 | 91 | TCCTCCCACAATCCTCAGTTTGCT | ATGGTTTGCCTCTCTCTCCTTGCT | Gain |
| Primer15 | 15 | 32,990,312-32,990,457 | 146 | AGCAAGGACAGTGGCTTGTAGGAA | TGGCTGAAGAGTTCTCCTGTGCTT | Gain |
| Primer16 | 15 | 33,019,515-33,019,687 | 173 | TGGAGGAGACATGAGGCCATGAAA | TCTGAACTGCACCAAAGGAGACCA | Gain |
| Primer17 | 15 | 33,028,577-33,028,722 | 146 | AGAATCCTCAGCATTGGAGAGCCA | TGATGTGTGTGTGTGAGTGGAGGT | Gain |
| Primer18 | 16 | 57,022,324-57,022,437 | 114 | GGCAGATAATGACCCTCAACTC | GACCACACCCAAATCCCATAA | Gain |
| Primer19 | 18 | 33,247,170-33,247,345 | 176 | TCTGGCACAGTGAAACACTGCCTA | AGCTAGCCAGAGAATGTTGCTGGA | Loss |
| Primer20 | 18 | 47,723,553-47,723,654 | 102 | CAACTGAACCCACCGCTATAA | CCTTCCAGAAGTGCTGATGAA | Gain |
| Primer21 | 19 | 23,000,152-23,000,266 | 115 | GCTACAACAGCCAACCTTGCATGA | AATGAGGGAACTGGATGTGAGCGA | Gain |
| Primer22 | 19 | 23,040,969-23,041,076 | 108 | CATCATTGGCTGTTGTTCTGCGGT | ATGATCCCAAGACAGCTGGTGACA | Gain |
| Primer23 | 19 | 23,138,778-23,138,905 | 128 | TCACAGGCAGGGAAGTACAGGTTT | CAACACGTGCTTCTTGTTCCAGCA | Gain |
| Primer24 | 19 | 23,198,429-23,198,531 | 103 | TGACCACATCTGCTCCAAAGACCT | TCATGCCTTGAGACTTCCCTGTGT | Gain |
| Primer25 | 19 | 23,254,458-23,254,566 | 109 | AATGGCCTTGTCCCAGAGACTGAA | CTTGCAGGAACAAGCAGTTGGTGT | Gain |
| Primer26 | 19 | 23,301,491-23,301,612 | 122 | AACTCAGGTTCCACCTCACATGCT | ACAGGCTGGCAACAGTTGTGAATG | Gain |
| Primer27 | 19 | 23,344,761-23,344,911 | 151 | TCACTCAGTGTGTCTGGTGTTGCT | AAGCATCAAAGTGGCCAGAACAGG | Gain |
| Primer28 | 22 | 46,347,041-46,347,145 | 105 | CCTTGATGTGCCCTCCTTATT | GGAAGCATTCTCAGGAGTGTAA | Gain |
| Primer29 | 23 | 23,626,405-23,626,534 | 130 | AGCTAATGTGCTTCAGTCCAGGGT | TGAGAATACGGCCCAAGGGAAAGT | Gain |
| Primer30 | 25 | 48,082,247-48,082,434 | 188 | TGAGTGTGTGGATGGGCTTAGGTT | TGCAAGTGAAAGGTGGTTCTTGGC | Gain |
| Primer31 | 32 | 41,671,312-41,671,452 | 141 | ATAATGAGCGGCTTGGAGGTAGCA | TGTGGTCTGGGCCCTTTCACTTAT | Gain |
| Primer32 | 32 | 41,708,519-41,708,599 | 81 | TGCAGTTGCTCTGTTAGTGTCCCT | AGCTCACAGGAAGTTGGAGCAGAA | Gain |
| Primer33 | X | 1,781,765-1,781,916 | 152 | ACACCGTGGGTGTTTCCTGTATCA | ATCCAAACAGCGTGAGCATTCTGC | Gain |
| Primer34 | X | 3,035,569-3,035,686 | 118 | TGGGTTACTGACACCACCAAGTGT | GCAGCCCAAATTCTGGTTGCCTTA | Gain |
| Primer35 | X | 3,762,853-3,762,988 | 136 | TTGTCCACCAGGCTTGAACTCAGA | TCCAAGGTGCATGGTTCTCACGTA | Gain |
| Primer36 | X | 4,758,978-4,759,064 | 87 | ATCCAGGTGCTCTACAAACTGCCT | ATGGGCAATTCCTGCAAACAGGTC | Gain |
| Primer37 | X | 4,871,284-4,871,457 | 174 | TAGCTCCCAGGCAAGCTATGTGTT | AGGAGGTCAGCCATGTTGGAGAAA | Gain |
| Primer38 | X | 6,592,670-6,592,827 | 158 | ACACACAGTTCTCTAGCAGCCCAT | AAAGATGTCACAAAGCAGCCACGG | Gain |
| Primer39 | X | 6,602,322-6,602,443 | 122 | TGGTCCAGAAGTAAACGTGACCCA | TCCTGAGCGCCATGACAATATCCA | Gain |


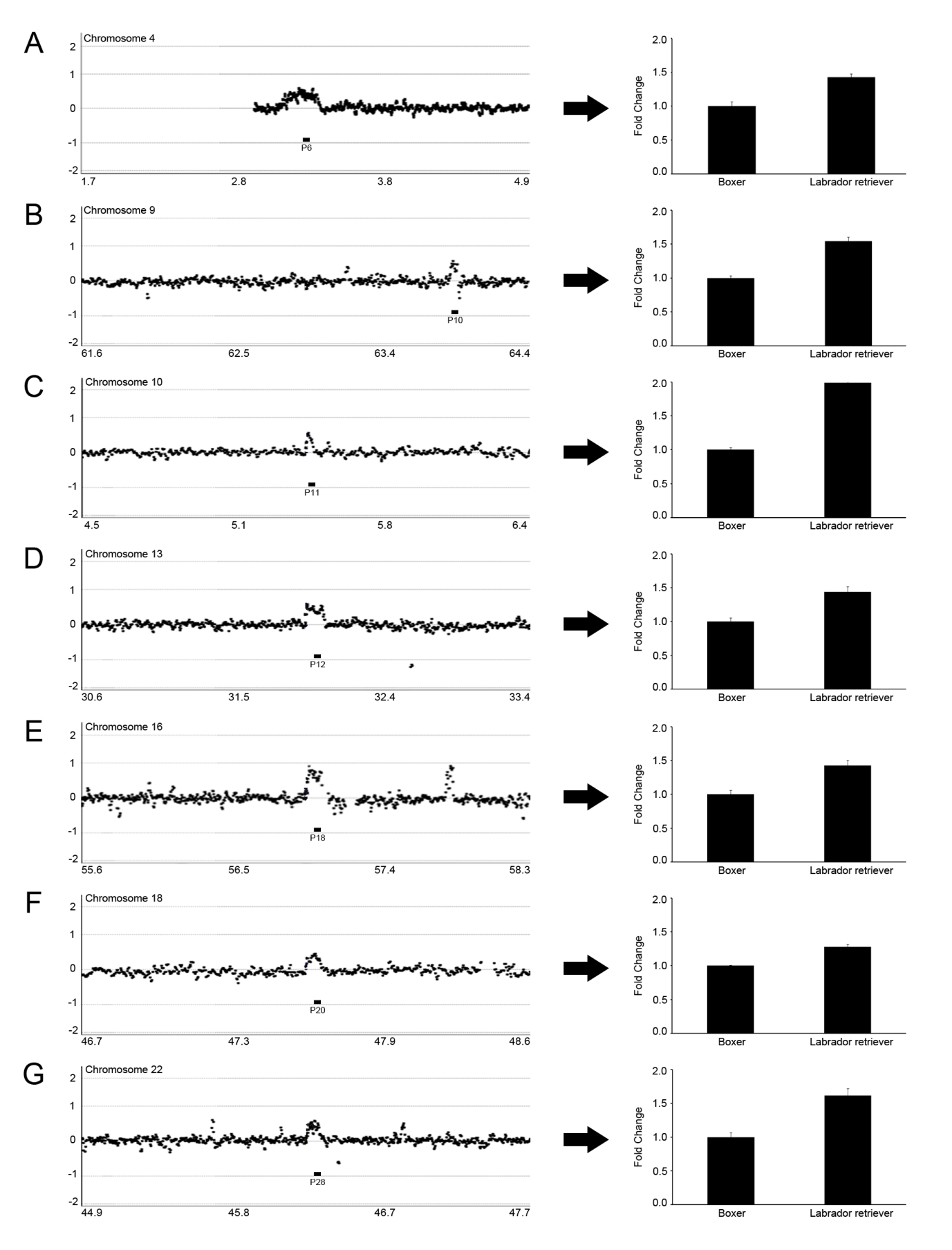


**Figure S1**. **Novel CNVs identified between a Labrador retriever and a Boxer, and qPCR validation.** Left, Log_2_ratio plot around the 7 novel CNV regions (A-G). One novel CNV on chromosome 4 (A) is also included in the 9 validation targets. The X-axis represents genomic position (Mb) and the Y-axis represents signal intensity ratios (Labrador retriever/Boxer) on log_2_ scale. Black bar represents primer position for qPCR validation. Right, Comparison of the copy number between Boxer and Labrador retriever by genomic qPCR. The Y-axis represents fold change based on boxer as calibrator.


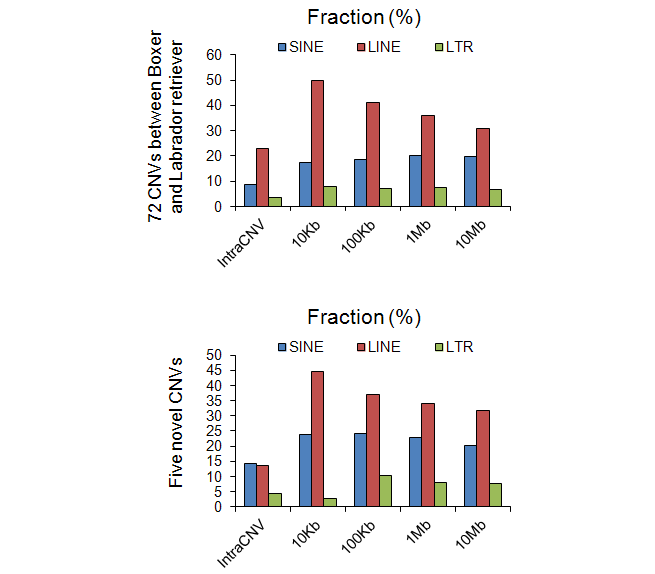


**Figure S2. Genomic fraction of the repetitive sequence elements.** We measured the genomic fraction (fraction of retroelements per kb) of SINE (short interspersed elements), LINE (long interspersed elements) and LTR (long terminal repeats) within (intraCNV) or in the vicinity of the CNVs (up to 10Mb).


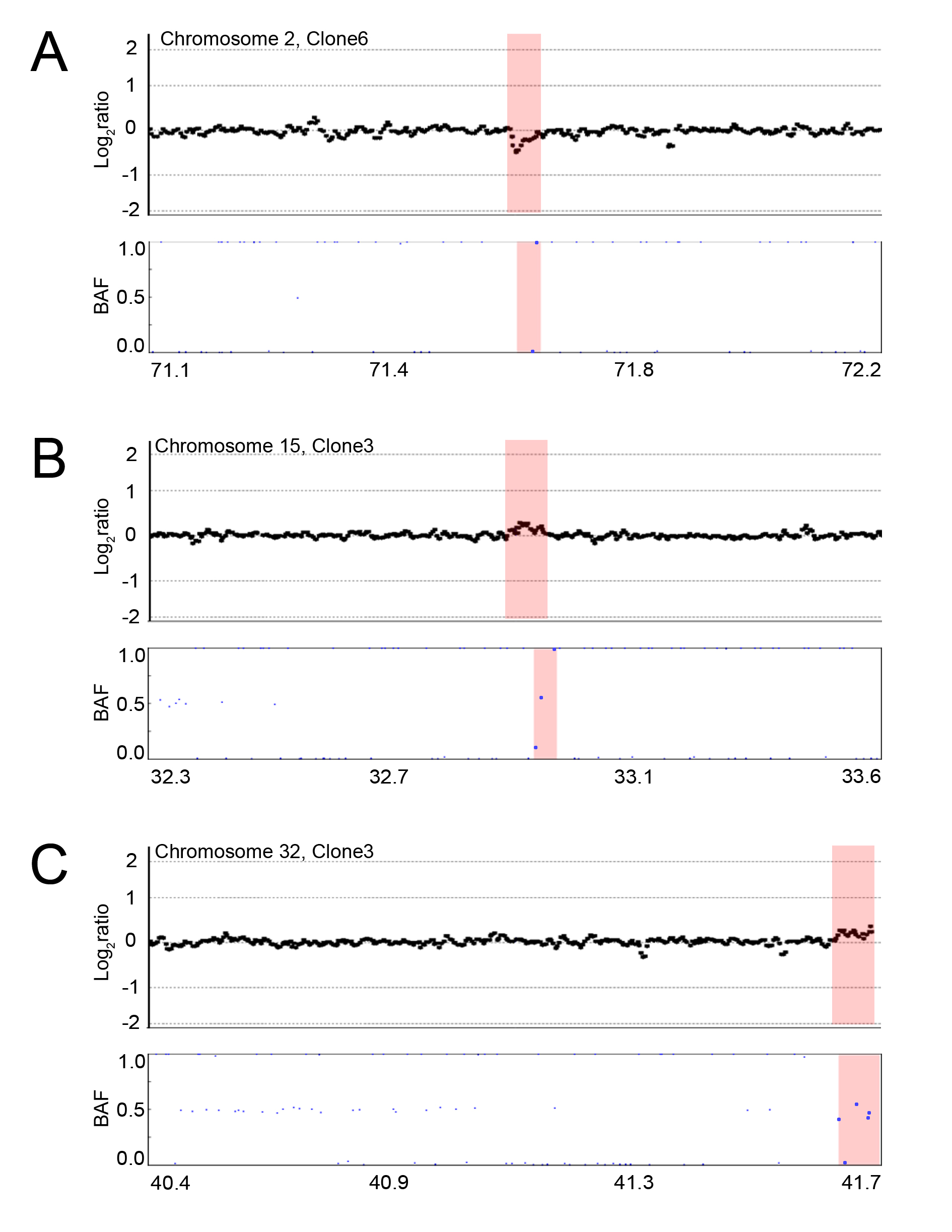


**Figure S3**. **B allele profiles around the three small-sized de novo CNVs.** Upper panel, Log2ratio plot on chromosome 2 (36.1 Kb) (A), chromosome 15 (42.2 Kb) (B) and chromosome 32 (76.4 Kb) (C). Red box represents CNV region. The X-axis represents genomic position (Mb) and the Y- axis represents signal intensity ratio on a log2 scale. Lower panel, B allele plot of the same genomic locus. Red box represents the same CNV region. Only two to five SNP probes were located in copy number variation region, therefore interpretation of LOH based on the BAF pattern was unavailable.
